# Healthcare resource utilisation and associated costs after low-risk pulmonary embolism: pre-specified analysis of the Home Treatment of Pulmonary Embolism (HoT-PE) study

**DOI:** 10.1007/s00392-023-02355-5

**Published:** 2024-01-03

**Authors:** Ioannis T. Farmakis, Klaus Kaier, Lukas Hobohm, Katharina Mohr, Luca Valerio, Stefano Barco, Stavros V. Konstantinides, Harald Binder

**Affiliations:** 1 Center for Thrombosis and Hemostasis, University Medical Center of the Johannes Gutenberg University, Langenbeckstrasse 1, 55131 Mainz, Germany; 2 Institute of Medical Biometry and Statistics, Faculty of Medicine, University of Freiburg, Freiburg im Breisgau, Germany; 3 Department of Cardiology, University Medical Center of the Johannes Gutenberg University, Mainz, Germany; 4 Department of Angiology, University Hospital Zurich, Zurich, Switzerland; 5 Department of Cardiology, Democritus University of Thrace, Alexandroupolis, Greece

**Keywords:** Pulmonary embolism, Low-risk, Home treatment, Cost-of-illness, Productivity loss, Healthcare resource utilisation

## Abstract

**Background:**

Pulmonary embolism (PE) and its sequelae impact healthcare systems globally. Low-risk PE patients can be managed with early discharge strategies leading to cost savings, but post-discharge costs are undetermined.

**Purpose:**

To define healthcare resource utilisation and overall costs during follow-up of low-risk PE.

**Methods:**

We used an incidence-based, bottom–up approach and calculated direct and indirect costs over 3-month follow-up after low-risk PE, with data from the Home Treatment of Patients with Low-Risk Pulmonary Embolism (HoT-PE) cohort study.

**Results:**

Average 3-month costs per patient having suffered low-risk PE were 7029.62 €; of this amount, 4872.93 € were associated with PE, accounting to 69.3% of total costs. Specifically, direct costs totalled 3019.33 €, and of those, 862.64 € (28.6%) were associated with PE. Anticoagulation (279.00 €), rehospitalisations (296.83 €), and ambulatory visits (194.95 €) comprised the majority of the 3-month direct costs. The remaining costs amounting to 4010.29 € were indirect costs due to loss of productivity.

**Conclusion:**

In a patient cohort with acute low-risk PE followed over 3 months, the majority of costs were indirect costs related to productivity loss, whereas direct, PE-specific post-discharge costs were low. Effective interventions are needed to reduce the burden of PE and associated costs, especially those related to productivity loss.

**Graphical abstract:**

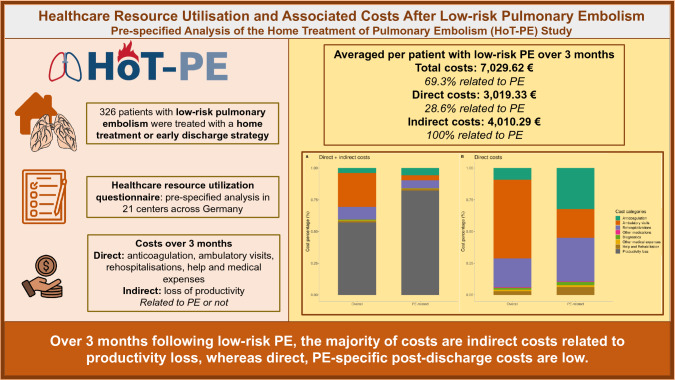

## Introduction

Pulmonary embolism (PE), a clinical manifestation of venous thromboembolism (VTE), constitutes a major burden for healthcare systems worldwide [1, 2]. The rising incidence rates of PE, reflecting the ageing of societies as well as decreasing mortality rates in the acute phase of PE result in an increasing population of patients surviving an episode of PE [3–5]. Those patients may suffer from persistently poor physical performance and may be faced with permanent or temporary inability to work in the aftermath of the index event. Moreover, a substantial percentage of patients report poor quality of life and/or present with clinical and functional post-PE impairment [6, 7]. Long-term sequelae as well as the ongoing risk for recurrent thromboembolic and treatment-related bleeding complications contribute significantly to the overall burden imposed by PE to patients and the society [8].

Based on the baseline risk stratification of the acute event, a population at low risk for early mortality can be defined [3]. Low-risk PE accounts for approximately 20–30% of all PE cases according to contemporary patient cohort studies [7, 9]. These patients, if they fulfil well defined clinical and social criteria, are potentially eligible for early discharge and ambulatory treatment. Such strategies, which appear to be feasible and safe as indicated by prospective multicentre studies, aim at reducing costs compared to a conventional duration of in-hospital management of acute PE [10, 11]. However, healthcare resource utilisation and the costs related to the ambulatory treatment of this ‘low-risk’ population over the first 3 months after the acute PE event have not been systematically studied. Therefore, the primary aim of the present study was to determine these costs using data from a prospective, single-arm management (cohort) study of patients with low-risk PE following an early discharge strategy [10, 12]. Further aims of the study were to dissect the main drivers of ambulatory or in-hospital long-term costs, and to identify subgroups of patients with excess costs among those with a low-risk PE event.

## Methods

We used an incidence-based, bottom–up approach to perform a cost-of-illness and healthcare resource utilisation analysis for low-risk PE treated with an early discharge strategy.

### Patient population and data collection

This was a pre-specified analysis of The Home Treatment of Patients with Low-Risk Pulmonary Embolism with the Oral Factor Xa Inhibitor Rivaroxaban (HoT-PE) trial [13]. Briefly, HoT-PE was a prospective, single-arm, phase 4 clinical study performed in seven countries which included patients with an objectively confirmed diagnosis of acute PE and absence of right ventricular (RV) dysfunction or overload. The primary objective of the study was to determine whether a strategy of initiation of anticoagulation with rivaroxaban followed by an early discharge (< 48 h) and continuation of treatment at home for at least 3 months after enrolment was effective and safe in patients with low-risk acute PE. The study was terminated early after a pre-defined interim analysis of 50% of the initially planned intention-to-treat trial population [10]. The institutional ethics review board of each participating site approved the study protocol and all patients provided informed consent for participation in the study. For the purpose of this pre-specified cost and healthcare resource utilisation analysis only centres from Germany were included (*n* = 21 centres).

### Study perspective, time horizon and definition of costs

This cost and healthcare resource utilisation analysis was performed from a societal perspective and included direct and indirect costs, such as those paid by insurances or patients, and loss of productivity costs. The time horizon over which costs were evaluated was the 3 months after the index event corresponding the treatment and follow-up duration of the HoT-PE study. Direct costs included those related to anticoagulation (in this case, the study drug rivaroxaban), ambulatory visits to medical professionals, rehospitalisations, formal or informal help (professional nursing, stationary or ambulatory rehabilitation, and household help, respectively), and other medical expenses (e.g. physiotherapy, compression stockings, breathing training devices) during 3-month follow-up. Indirect costs included costs related to productivity loss (hours of work lost) specifically due to PE were assigned only among patients previously employed, while they were estimated using the human-capital method. Because the duration of productivity loss was recorded as a categorical variable (≤ 6 weeks vs > 6 weeks), patients reporting ≤ 6 weeks productivity loss were assumed to have suffered 3 weeks of productivity loss, whereas patients reporting > 6 weeks productivity loss were assumed to have suffered 9 weeks of productivity loss. Costs were determined to be related to PE (disease-specific) by the investigators of each site conducting the questionnaire; productivity loss was assumed to be disease-specific following the acute PE index event. Because of the 3-month timeframe of this analysis no discounting was applied. The monetary valuation of intangible losses was not estimated.

### Cost inputs and calculations

Taking into account that the totality of patients included in this analysis were living in Germany, cost units for the direct costs were calculated using standardised unit costs. In detail, cost inputs from Bock et al. were used for rehospitalisation(s) [14]; all other direct costs as well as the costs of productivity loss (indirect costs calculated only for patients with employment prior to the event) were retrieved from the German Ministry of Health and large German insurance organisations. All cost inputs were expressed in 2021 Euros (€) adjusting for inflation. A detailed presentation of the cost inputs and sources is depicted in Table 1.

**Table 1:** Pulmonary embolism associated cost inputs adjusted for inflation and purchasing power parity (2021 Euros €)

Cost unit	Value (2021 €)
Anticoagulation	
Rivaroxaban, per day	3.1
Enoxaparin, per day	14.49
Tinzaparin, per day	15.1
Fondaparinux, per day	18.58
Medical ambulatory visits, per visit^a^	As described by the statutory health insurances per respective specialty
Hospitalisations, per inpatient day^b^	658.27
Diagnostic procedures, per examination^c^	
Chest X-ray	9.1
CT	65.19
MRT	117.14
Endoscopy/bronchoscopy	127.04
Lung scintigraphy	44.61
Echocardiography	27.3
Compression ultrasound	8.12
Rehabilitation clinic, per day^d^	135.25
Formal and informal help, per visit	15.0
Other medical expenses, per item	
Breathing training	20.0
Compression stockings	15.0
Physiotherapy^e^	26.7
Productivity loss, per day of absence^f^	343.95

Inflation was calculated with the use of https://www.inflationtool.com/euro, PPPs were taken from https://ec.europa.eu/eurostat/databrowser/view/prc_ppp_ind/default/table?lang=en

*CT* computed tomography, *MRT* magnetic resonance tomography

^a^Found in https://www.kbv.de/media/sp/Honorarbericht_Quartal_3_2020.pdf

^b^Found in [14]

^c^Found in https://www.kbv.de/media/sp/EBM_Gesamt_-_Stand_4._Quartal_2021.pdf

^d^As per https://onlinelibrary.wiley.com/doi/10.1111/jdv.17203

^e^Found in https://www.vdek.com/content/dam/vdeksite/vdek/themen_vertragspartner/Heilmittel-Hilfsmittel/20210801_Physiotherapie_Anlage_2__Verguetungsvereinbarung.pdf

^f^Based on data from the „Bundesanstalt für Arbeitsschutz und Arbeitsmedizin“ https://www.baua.de/DE/Themen/Arbeitswelt-und-Arbeitsschutz-im-Wandel/Arbeitsweltberichterstattung/Kosten-der-AU/Kosten-der-Arbeitsunfaehigkeit_node.html

During follow-up, total average costs per patient with low-risk PE comprised the cost categories described above. The proportion of costs associated with PE to the total costs for the overall costs, and for each cost category, were calculated and the result was regarded as excess costs due to PE. The association with PE was determined in a separate field for each cost component domain by the investigators responsible for the questionnaire completion. Pre-defined analyses were performed to explore differences between subgroups of: (i) age (≤ 65 vs > 65 years), (ii) sex (women vs men), (iii) presence of comorbidity (at least one of: active cancer, chronic obstructive pulmonary disease, chronic heart failure, coronary artery disease, diabetes mellitus), (iv) patient fragility (defined as at least one of: age > 75 years, creatinine clearance < 50 ml/min, or body mass index < 18.5 kg/m^2^ [15]). 95% confidence intervals for cost figures were calculated using generalised linear models with a log link and a gamma distribution. Missing data in a cost category were assumed as no utilisation and were replaced with zero € in case at least one question in the cost questionnaire was completed by the patient. All calculations were performed using Stata 17 (Stata Corp., Texas, USA) and figures were produced with R (the R Project for Statistical Computing, version 4.1.1).

## Results

A total of 326 patients with a mean age of 55.1 ± 16.6 (range 19–86) years completed the healthcare resource utilisation questionnaire and took part in the analysis. The baseline clinical characteristics of the patients are presented in Table 2. At least one comorbidity was present in 68/326 (20.9%) patients, while 56/326 (17.2%) patients were characterised as fragile.

**Table 2:** Baseline characteristics of the patients

Characteristic	*N* = 326
Demographics	
Age (years)	55.1 (16.7)
Women	152 (46.7%)
Body mass index (kg/m^2^)	28.0 (5.9)
Employed before PE	157/277 (56.7%)
Comorbidities	
Active cancer	16 (4.9%)
Chronic obstructive pulmonary disease	19 (5.8%)
Chronic heart failure	6 (1.8%)
Coronary artery disease	23 (7.1%)
Diabetes mellitus	24 (7.4%)
Creatinine clearance (ml/min)	80.5 (18.8)

Reported are mean (SD) for continuous and number (%) for categorical variables

**Fig. 1:**
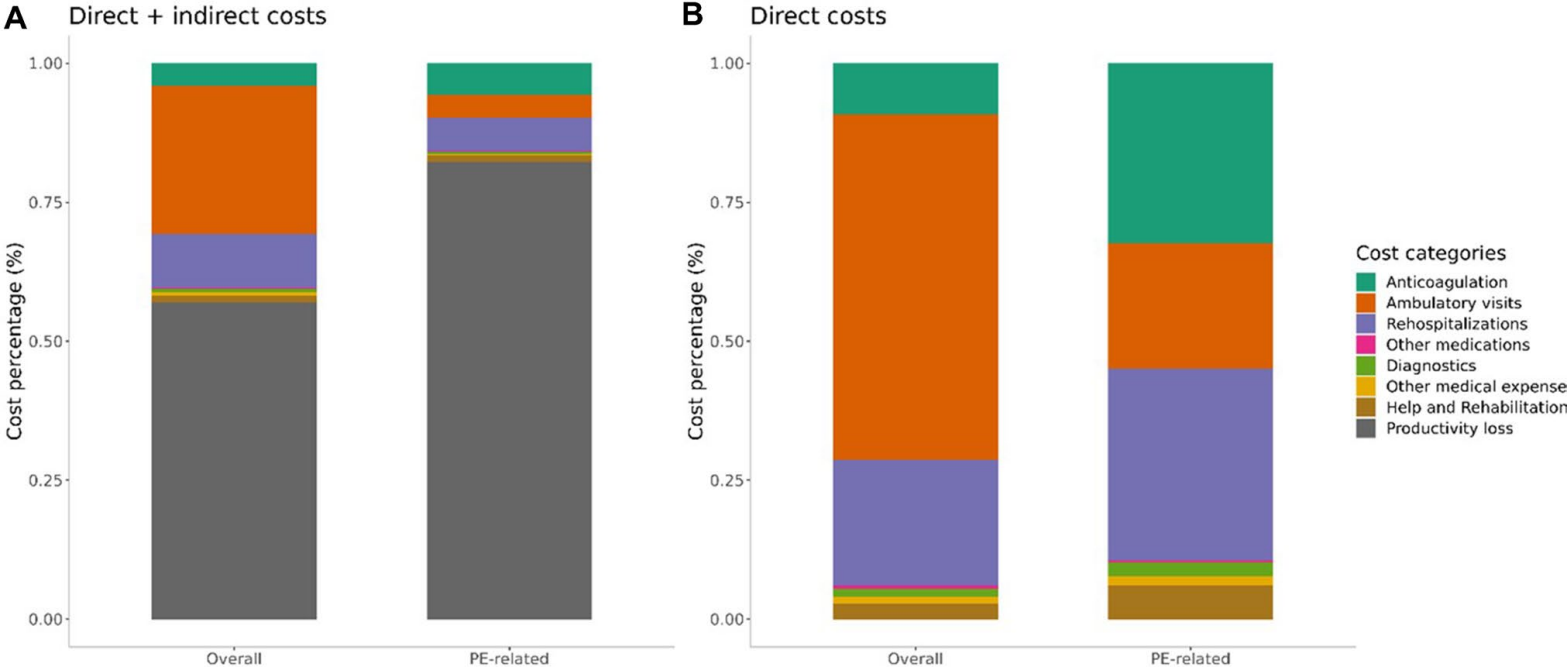
Proportion of cost components to the overall costs (**A**) and to only the direct costs (**B**)

The total average costs per patient with low-risk PE over 3-month follow-up were 7029.62 €; of this amount, 4872.93 € were related to the index acute PE event (and thus defined as excess costs due to PE), accounting to 69.3% of total costs. The percentage contribution of the different cost categories to the overall costs is shown in Fig. 1.

### Direct costs

The direct costs totalled 3019.33 €, and of those, 862.64 € were associated with PE (28.6%). The largest proportion of the costs associated with PE resulted from anticoagulation, rehospitalisations, and ambulatory visits (Fig. 1B). All patients received 90 days of anticoagulation with rivaroxaban after discharge accounting for an average cost of 279 € per patient. During the 3-month follow-up, 35 (8.89%) patients were hospitalised. Among those patients, the average number of hospitalisation days was 11.66 with an average cost of hospitalisation of 7675.42 €. Overall, the average (re)hospitalisation cost per patient included in the HoT-PE study was 682.51 € (296.83 € associated with PE).

In addition to hospitalisations, 313 (96.01%) patients visited a physician during the 3-month follow-up totalling to 16.24 ambulatory visits per patient; of them, 15.71% (2.55 per patient) ambulatory visits were associated with PE. The cost of ambulatory visits per patient included in the study was 1873.04 € (194.95 € associated with PE). The utilisation of diagnostic procedures, formal or informal help, rehabilitation, and other medical expenses are presented in Table 3, while the costs related to these measures are presented in Table 4.

**Table 3:** Health resource utilisation per low-risk PE patient over 3 months of follow-up

Health resource	Value	Value related to PE
Ambulatory visit, *n*/*N*	313/326 (96%)	207/326 (63%)
Number of ambulatory visits, mean (SD; range)	16.24 ± 1.03	2.55 ± 0.79
Hospitalized patients, * n*/*N*	29/326 (9%)	10/326 (3%)
Length of stay, mean ± SD	11.65 ± 22.02	14.7 ± 32.72
Formal and informal help since discharge of hospital, * n*/*N*	5/326 (1.5%)	2/326 (0.6%)
Period of help, days	28.65 ± 18.42	11.25 ± 0
Rehabilitation an inpatient setting, * n*/*N*	3/326 (0.9%)	3/326 (0.9%)
Rehabilitation days	38.5 ± 24.25	38.5 ± 24.25
Rehabilitation in an outpatient setting, * n*/*N*	3/326 (0.9%)	1/326 (0.3%)
Rehabilitation days	38.5 ± 24.25	10.5 ± 0
Disability due to disease or its treatment (productivity loss), * n*/*N*	115/326 (35%)	115/326 (35%)
Period of disability, days	33.05 ± 19.08	33.05 ± 19.08
Diagnostic procedures at follow-up, number of examinations		
CT	0.11 ± 0.36	0.05 ± 0.21
MRT	0.06 ± 0.25	0.02 ± 0.12
Endoscopy/bronchoscopy	0.07 ± 0.34	0.04 ± 0.23
Lung scintigraphy	0.00 ± 0.00	0.00 ± 0.00
Echocardiography	200/326 (61.4%)	200/326 (61.4%)
Compression ultrasound	149/326 (45.7%)	149/326 (45.7%)
Other medical expenses, items per patient		
Physiotherapy	0.91 ± 4.53	0.22 ± 1.95
Compression stockings	0.57 ± 0.88	0.50 ± 0.83
Breathing training device	0.02 ± 0.15	0.01 ± 0.11

*CT* computed tomography, *MRT* magnetic resonance tomography, *PE* pulmonary embolism

**Table 4:** Average cost components per low-risk PE patient over 3 months of follow-up

Cost component	Costs overall (2021 €)	Costs related to PE (2021 €)
Direct costs		
Costs related to anticoagulation at follow-up	279.00	279.00
Costs related to ambulatory visits	1873.04 ± 706.05	194.95 ± 243.67
Costs related to rehospitalisation	682.51 ± 4784.31	296.83 ± 3955.16
Costs related to medications other than anticoagulation	19.06 ± 50.10	3.46 ± 16.91
Costs related to additional examinations (e.g. CT, MRI, etc.)	44.98 ± 49.22	21.78 ± 35.17
Costs related to other medical expenses (e.g. physiotherapy, compression stockings, breathing training)	33.90 ± 123.14	13.8 ± 55.26
Costs related to formal (professional help, e.g. nursing and/or rehabilitation centre) and informal (e.g. household) help	86.85 ± 644.39	52.82 ± 582.52
Indirect costs		
Costs related to productivity loss (hours of work lost)	4010.29 ± 6686.37	4010.29 ± 6686.37
Overall costs	7029.62 ± 8371.18	4872.93 ± 7841.96

### Indirect costs

The indirect costs, notably those due to loss of productivity, totalled 4010.29 € and made up the majority of overall costs (57.0%; Fig. 1A), especially when considering only the costs related to the index PE event (82.3%) (Fig. 1B). Employed before the index event were 157/277 (56.7%) patients (49 patients had an unknown employment status) and of these, 115 (73.2%) reported inability to work after the index PE hospitalisation for a mean of 33.05 days.

### Direct costs in the follow-up of low-risk PE across subgroups

**Fig. 2:**
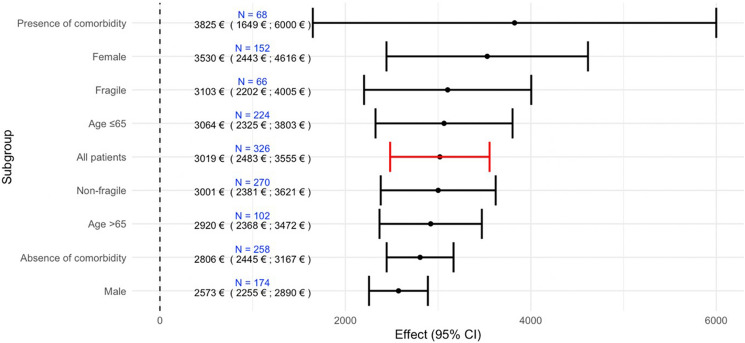
Direct costs in the 3-month follow-up of low-risk PE across selected subgroups of patients

Concerning direct costs, these were higher for patients with comorbidities (3825.33 € vs 2806.90 € for patients without comorbidities), female patients (3530.23 € vs 2573.03 € for male patients) mainly because of the higher costs of hospitalizations during follow-up (Fig. 2). There were no major differences based on fragility (3103.82 € for fragile vs 3001.81 € for non-fragile patients) or age (3064.35 € for age ≤ 65 years vs 2920.48 € for age > 65 years).

## Discussion

In this cost analysis of low-risk PE, the overall costs over a 3-month follow-up period, summed up to 7000 €, with costs specifically related to PE representing accounting for 70% of the total costs. The largest proportion of costs were made up from indirect costs related to productivity loss and direct disease specific costs were low.

While previous analyses have focussed on the cost effectiveness of a home treatment strategy for low-risk PE [16], this is the most comprehensive analysis to focus on the healthcare resource utilisation and associated costs in the follow-up of low-risk PE. A previous analysis of the YEARS study aimed to quantify the average total healthcare costs during a 3-month follow-up period and concentrated on the comparison of patients treated as outpatients versus those hospitalised, estimating a 1483 € net cost reduction with a home treatment strategy [17]. However, that analysis was planned post hoc and provided little information regarding costs related to follow-up, including only PE-related admissions and outpatient visits; there was also no information regarding indirect costs. On the contrary, our analysis was pre-specified, and a prospectively defined questionnaire covering all aspects of post-PE healthcare resource utilisation was used, including information regarding absence from work following to the index event.

As is evident from our analysis, costs related to productivity loss comprise the majority of disease-specific costs during the follow-up of low-risk PE. In an analysis of the PREFER in VTE registry, after applying an 80-day friction period (meaning that patients absent from work for a period greater than 80 days were assumed to be replaced, thus, no longer imposing costs on the society), the indirect costs made up 42–49% of total costs after PE with a time horizon of 12 months [8]. Accordingly, also with a time horizon of 12 months, an analysis of Danish nationwide data showed that 47.3% of total costs after a venous thromboembolism event were attributed to productivity loss; interestingly, the attributable percentage of productivity loss was larger after deep vein thrombosis (52.9%) than after PE (42.6%) [18]. If expressed as proportion of the total costs, indirect costs would be more significant over the first 3 months, given the smaller amount of direct costs (smaller number of chronic PE complications due to the shorter follow-up time), while the indirect costs would be unaffected since the loss of productivity due to an acute event begins by definition on the first day of follow-up. In that sense, the Danish nationwide data appear to be in agreement with the results of our analysis. Moreover, given the similarity of percentages with the PREFER in VTE study, which included patients with PE irrespective of disease severity, and the Danish deep vein thrombosis population, we can hypothesise that the severity of the underlying venous thromboembolism (or PE) event may not be the only factor that heavily influences the return or not to work. Although a more severe acute PE event is associated with higher likelihood of persistent cardiopulmonary exercise limitation after PE [19], more than half of patients after PE with neither severe PE nor major comorbidities are found to be suffering from exercise limitation [19, 20]. In addition, impaired quality of life after PE may not be necessarily related to the baseline risk stratification [21], and additionally, anxiety and depression are common among post-PE patients [22]. Optimal follow-up strategies, as well as reassurance at discharge for the low probability of post-discharge complications should be offered to all patients after low-risk PE in an attempt to reduce the societal costs attributed from productivity loss [23]. Rehabilitation outpatient programmes have shown some promise of efficacy by increasing functional capacity and quality of life in patients with post-PE dyspnoea; however, the evidence available so far comes from single-arm uncontrolled studies [24, 25]. The effectiveness of such strategies, also in terms of helping patients return more quickly to their daily activities and their work, remains yet to be confirmed by randomised controlled trials.

Previous studies performed in patients with PE also provide an opportunity for comparison of the direct costs related to PE with the results of this analysis, which exclusively comprised a low-risk patient population. The YEARS analysis estimated the 3-month cost of PE-related admissions and outpatient visits at 422 € (prices reflect Netherlands 2018 €; this corresponds to 406 German 2021 €, adjusting for inflation and purchase power parity) in patients treated at home and at 313 € (301 German 2021 €) in patients treated at hospital [17]. In our study, the respective amount was slightly higher at 492 €. Furthermore, we have previously reported a cost of illness analysis of PE comprising patients across the spectrum of risk stratification using data from the PREFER in VTE registry [8]. Disease-specific direct costs for the first year of follow-up after an incident PE case were estimated at 2370–2650 € (prices are EU-27 2020 €, and correspond to 2625–2936 German 2021 € adjusting for inflation and purchase power parity), while the respective amount concerning PE-associated costs in our study was 862.6 € with a 3-month time horizon. Direct costs in that study were mainly driven by anticoagulation, ambulatory visits, and rehospitalisations as it is also the case in the present study. Current guidelines recommend therapeutic anticoagulation for ≥ 3 months for all patients with PE and the decision for the extension of anticoagulation should be based only on the presence of venous thromboembolism risk factors and by no reason to the baseline risk stratification [3]. Moreover, a routine clinical ambulatory evaluation is also recommended for all patients 3–6 months after the acute PE episode, while further diagnostic evaluation is not based on the severity of the index event, but rather on the clinical evaluation at the time of the follow-up [3]. Lastly, a previous healthcare resource use analysis from the PREFER in VTE showed a linear course of rehospitalisations across 12 months of follow-up [26]. Taking into account the abovementioned information, we can explain the lack of difference in direct costs attributable to PE in patients with low-risk compared to the overall PE patient population.

### Limitations

There are some limitations of this analysis that need to be mentioned. First, these results apply only to the German population, since participants from other countries were not included. Thus, the extrapolation of results to other countries may be applicable. Second, all patients received rivaroxaban. In general, the costs of different direct oral anticoagulants do not differ substantially. Although, the percentage of patients with routine use of low molecular weight heparin or fondaparinux is low among patients with PE, the price difference is substantial and this would slightly drive the costs up. On the other, vitamin K antagonists are generally cheaper than rivaroxaban, they entail, however, the adjunctive cost of routine international normalised ratio measurements and also currently used post-discharge in a minor subset of patients [8, 9]. Third, we were not able to precisely quantify the hours of work lost during follow-up, or the amount of reduced time of work once the patients returned to work [26], because the variable was captured as a dichotomized variable in the questionnaire provided to the patients, therefore, the indirect costs consist an approximation. However, our findings align with previous studies and support the validity of our results. Fourth, the definition of costs as being related to PE was not externally adjudicated. Fifth, we did not have access to the actual costs or reimbursements provided during follow-up. Instead, the patients were retrospectively asked to provide the number of hospitalisation days and outpatient visits, and standardised unit costs were applied to turn those visits into cost estimates. The individual diagnosis-related groups (DRG’s) of the respective follow-up hospitalisation were not recorded, nor was the specific reason for rehospitalization. Lastly, it was not feasible to estimate intangible losses, which is a typical limitation of cost-of-illness analyses that has conventionally been challenging to overcome.

## Conclusions

Over a 3-month follow-up period after a low-risk PE event the overall average costs per patient amounted to 7000 €. A significant proportion of these costs were related to PE representing excess costs due to the index event, accounting for 70% of the total costs, while indirect costs related to productivity loss constituted the largest proportion of costs, when direct disease-specific costs were found to be low. These findings highlight the need for effective interventions that can reduce the burden of PE and its associated costs, particularly those related to productivity loss.
